# Somatic mutations in salivary duct carcinoma and potential therapeutic targets

**DOI:** 10.18632/oncotarget.18173

**Published:** 2017-05-25

**Authors:** Timothy K. Khoo, Bing Yu, Joel A. Smith, Angus J. Clarke, Peter P. Luk, Christina I. Selinger, Kate L. Mahon, Spiridoula Kraitsek, Carsten Palme, Michael J. Boyer, Marcel E. Dinger, Mark J. Cowley, Sandra A. O’Toole, Jonathan R. Clark, Ruta Gupta

**Affiliations:** ^1^ Central Clinical School, The University of Sydney, Australia; ^2^ Department of Medical Genomics, Royal Prince Alfred Hospital, Sydney, Australia; ^3^ The Sydney Head and Neck Cancer Institute, Chris O’Brien Lifehouse, Sydney, Australia; ^4^ Department of Tissue Pathology and Diagnostic Oncology, Royal Prince Alfred Hospital, Sydney, Australia; ^5^ The Department of Medical Oncology, Chris O’Brien Lifehouse, Sydney, Australia; ^6^ Kinghorn Cancer Centre and Garvan Institute of Medical Research, Darlinghurst, Sydney, Australia; ^7^ South West Clinical School, University of New South Wales, Sydney, Australia

**Keywords:** salivary duct carcinoma, somatic mutation analysis, targeted therapies, androgen receptor, HER2

## Abstract

**Background:**

Salivary duct carcinomas (SDCa) are rare highly aggressive malignancies. Most patients die from distant metastatic disease within three years of diagnosis. There are limited therapeutic options for disseminated disease.

**Results:**

11 cases showed androgen receptor expression and 6 cases showed HER2 amplification. 6 Somatic mutations with additional available targeted therapies were identified: *EGFR* (p.G721A: Gefitinib), *PDGFRA* (p.H845Y: Imatinib and Crenolanib), *PIK3CA* (p.H1047R: Everolimus), *ERBB2* (p.V842I: Lapatinib), *HRAS* (p.Q61R: Selumetinib) and *KIT* (p.T670I: Sorafenib). Furthermore, alterations in *PTEN*, *PIK3CA* and *HRAS* that alter response to androgen deprivation therapy and HER2 inhibition were also seen.

**Materials and Methods:**

Somatic mutation analysis was performed on DNA extracted from 15 archival cases of SDCa using the targeted Illumina TruSeq Amplicon Cancer Panel. Potential targetable genetic alterations were identified using extensive literature and international somatic mutation database (COSMIC, KEGG) search. Immunohistochemistry for androgen receptor and immunohistochemistry and fluorescent *in situ* hybridization for HER2 were also performed.

**Conclusions:**

SDCa show multiple somatic mutations, some that are amenable to pharmacologic manipulation and others that confer resistance to treatments currently under investigation. These findings emphasize the need to develop testing and treatment strategies for SDCa.

## INTRODUCTION

Salivary duct carcinoma (SDCa) is a highly aggressive primary salivary gland malignancy associated with poor prognosis accounting for 10–12% of salivary gland malignancies [1, 2]. Currently, surgical resection of the primary tumor, neck dissection, and adjuvant radiation with or without platinum-based chemotherapy forms the mainstay of treatment. Despite this aggressive multimodal approach, the majority of patients with SDCa die within three years of diagnosis due to distant metastatic disease [2, 3]. This highlights the need for effective systemic therapy that can produce a sustained response.

The wide availability of massive parallel sequencing technology has revealed the somatic mutational landscape of many malignancies [4–7]. The identification of driver mutations and downstream effector targets has allowed development of targeted therapies that have revolutionized the treatment of subsets of selected malignancies such as breast, melanoma and pulmonary adenocarcinoma, often with lower toxicity than conventional chemotherapy [5–8]. Identifying effective targeted therapies in rare aggressive malignancies, such as SDCa, is particularly challenging, as well-powered clinical trials are not feasible. The use of targeted therapies in SDCa remains experimental, with variable response rates [5, 9–10]. Alternatives to traditional randomized controlled trials are needed, including targeted gene panel analysis of retrospective archival cohorts, with consideration of agents targeted at specific mutations with proven benefit in more common cancers. This approach has the potential to advance treatment and improve patient outcomes in this rare but lethal tumor [11–14].

This study aims to identify and analyze clinically relevant somatic mutations in SDCa that are amenable to targeted therapy. Moreover, we aim to identify somatic mutations that may alter response to these therapies. The data generated by this study can be used as a starting point for preclinical and clinical studies investigating targeted systemic therapy options for SDCa.

## RESULTS

### Cohort characteristics

The final cohort included 15 cases of SDCa where DNA extracted from retrospective FFPE tissues passed quality control. The cohort included 14 males and 1 female with a median age of 58 years (range 41–74). The median follow-up was 21.5 months (range 4–78 months), during which time 8 (53.3%) patients died of SDCa. Regional metastases were seen in 10 patients and two patients developed distant metastases to the lung, bone and / or brain. All patients were treated with local resection and adjuvant radiotherapy. In addition, nine patients underwent a neck dissection and five patients received adjuvant platinum-based chemotherapy and one patient is currently receiving Trastuzumab (Table 1). There were 11 patients positive for AR expression and of these 6 patients also demonstrated HER2 amplification (Table 2). None of the tumors demonstrated estrogen or progesterone receptor expression.

**Table 1:** Clinical features, treatment and follow up

Case No.	Age (y)	Sex	Presentation	Treatment	Follow-up (months)
1	63	M	NK	Submandibular gland excision and RT	DOD (37)
2	56	M	Submandibular mass	Submandibular gland excision, SND (I), and RT	AWD (54)
3	52	M	NK	Parotidectomy and RT	DOD (38)
4	72	F	Parotid mass	Total parotid, SND, RT, and CRT (Doxorubicin and Cyclophosphamide)	NED (4)
5	58	M	Parotid mass	Total parotid, SND (II), and RT	NED (78)
6	50	M	Parotid mass, facial weakness	Radical parotid, SND (II, III, V), RT, and CRT (Carboplatin)	DOD (16)
7	54	M	Parotid mass	Total parotid, MRND, RT, and CRT (Cisplatin then Paclitaxel)	DOD (25)
8	41	M	Sublingual mass	Sublingual gland excision and RT	NED (52)
9	63	M	Parotid mass	Superficial parotid, SND (II-III), RT, CRT (Cisplatin)	DOD (13)
10	64	M	NK	Parotidectomy and RT	AWOD (18)
11	58	M	Parotid mass	Total Parotid, RT, and CRT (Cisplatin then Paclitaxel and Trastuzumab)	DOD (28)
12	65	M	Parotid mass	Superficial parotid and RT	NED (14)
13	74	M	Submandibular mass	Submandibular gland excision, SND (I), RT, and CRT (Cisplatin)	NK
14	53	M	Lung metastases	Radical parotid, RND, and RT	DOD (6)
15	59	M	Parotid mass	Superficial parotid, SND (II), and RT	DOD (9)

Abbreviations: RND, radical neck dissection; RT, radiotherapy; MNRD, modified radical neck dissection; CRT, chemotherapy; SND, selective neck dissection; NK, not known; DOD, died of disease; AWD, alive with disease; AWOD, alive with other disease; NED, alive with no evidence of disease; DUC, died of unrelated cause.

**Table 2:** Clinico-pathological features of the salivary duct carcinoma cohort

Case no.	Tumor size	LVI	PNI	Margins	Stage	GATA3	AR Expression	HER2 FISH
1	48 mm	Not seen	Not seen	0.6 mm	T3Nx	+	−	Non-amplified
2	22 mm	Not seen	Not seen	0.2 mm	T2N0	+	+	Non-amplified
3	25 mm	Present	Not seen	Involved	T2N2b	+	+	Amplified
4	20 mm	Present	Not seen	Involved	T2N2b	+	+	Non-amplified
5	42 mm	Present	Not seen	1 mm	T3N2b	+	+	Non-amplified
6	17 mm	Extensive	Extensive	Involved	T4aN2b	+	+	Amplified
7	15 mm	Extensive	Present	Involved	T1N2b	+	+	Non-amplified
8	40 mm	Present	Present	Involved	T2Nx	+	+	Amplified
9	23 mm	Extensive	Present	Involved	T4aN2b	+	+	Amplified
10	64 mm	Extensive	Present	Involved	T4aN2b	+	+	Amplified
11	42 mm	Present	Present	0.3 mm	T3N2b	+	+	Non-amplified
12	50 mm	Not seen	Present	Involved	T3Nx	+	+	Amplified
13	15 mm	Present	Present	0.7 mm	T1N0	+	−	Non-amplified
14	40 mm	Extensive	Present	Involved	T2N2bM1	+	−	Non-amplified
15	35 mm	Extensive	Not seen	Involved	T2N2bM1	+	−	Non-amplified

Abbreviations: LVI, lymphovascular invasion; PNI, perineural invasion; N/A, not applicable; AR, androgen Receptor; GATA3, GATA binding protein 3; HER2. human epidermal growth factor receptor 2.

### Somatic mutations in SDCa

The mean coverage of 15 cases was 6087 (± 1848) with 95.1% (± 2.6%) uniformity of coverage at 0.2X of mean. Significant heterogeneity was present in terms of the number of somatic mutations present per patient (Mean = 19, range 1–154) A total of 289 non-synonymous mutations were identified in the 48-gene panel in 14 patients; after exclusion of a single patient where normal tissue was not available for comparison. These included 250 (86.5%) missense mutations, 14 (4.8%) nonsense mutations, 13 (4.5%) small indel mutations and 11 (3.8%) splice site changes.

**Figure 1:**
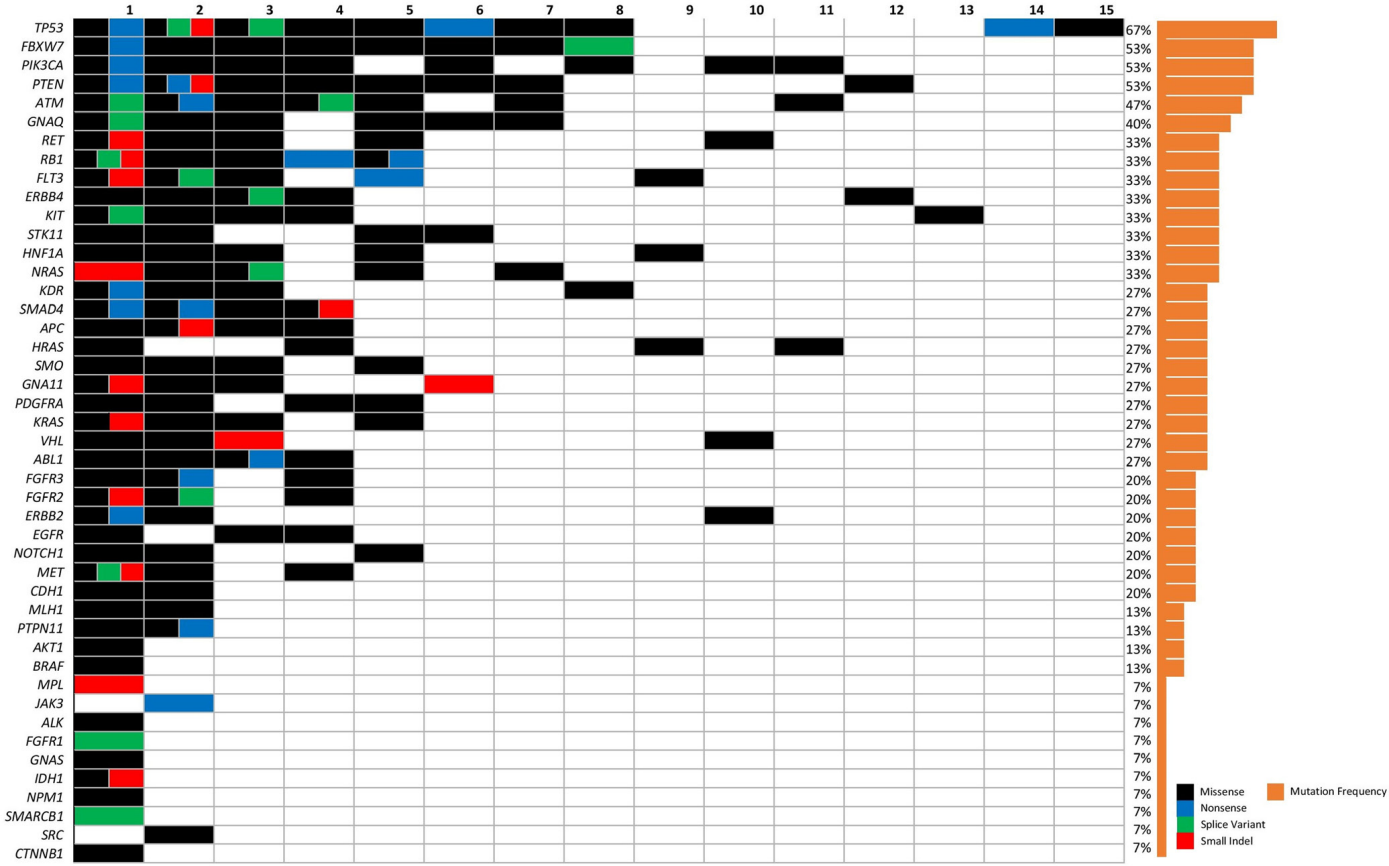
Frequency of somatic mutations found in the SDCa cohort The columns represent individual patients and rows represent specific type of mutation and relative frequency. Color legend of the aberrations represented including missense (black), nonsense (blue), splice variant (green) and small indel (red). Cases with more than one aberration are represented by a split cell with multiple colors.

The most commonly mutated genes in the cohort were *TP53* (*n* = 10), *PIK3CA* (*N* = 8), *PTEN* (*N* = 8), *FBXW7* (*n* = 8), *ATM* (*N* = 7), *GNAQ* (*N* = 6), and *HRAS* (*N* = 4) (Figure 1), (Supplementary Table 1).

**Figure 2:**
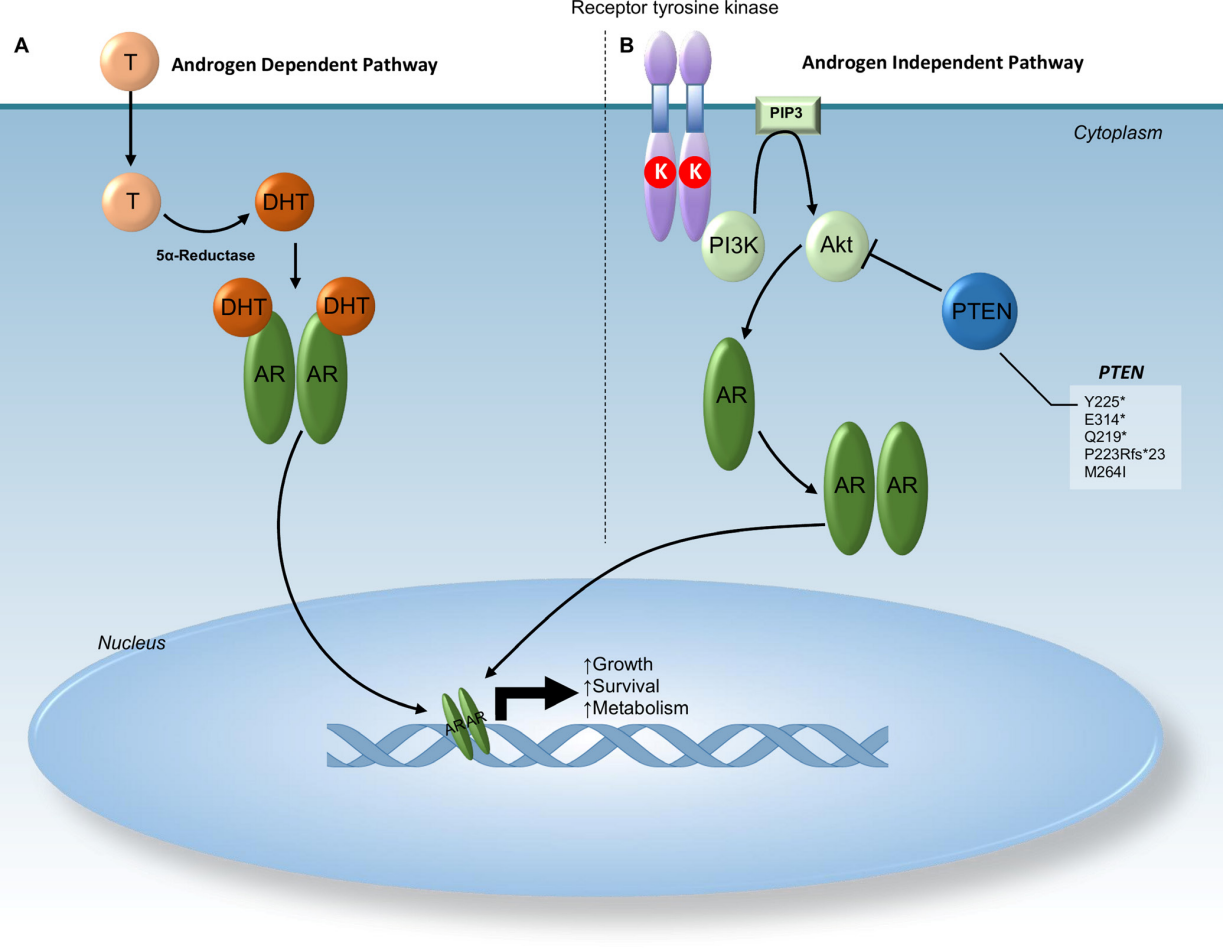
Schematic representation of androgen receptor pathway Section (**A)** demonstrates activation of androgen receptor (AR) by testosterone (T) under normal conditions. Signals from dihydrotestosterone cause dimerization of AR and relocation to the nucleus initiating metabolic activities and growth. Section (**B)** demonstrates activation of AR mediated by tyrosine kinase receptors. Dimerization of the tyrosine kinase receptor leads to activation of PI3K and phosphorylation of AKT. Phosphorylated AKT can then cause dimerization of AR leading to relocation of AR to the nucleus initiating metabolic activities and growth. Normally functional PTEN inhibits the AR dimerization initiated by phosphorylated AKT. Truncation and frameshift mutations in PTEN as seen in this cohort lead to the loss of this PTEN inhibition. Thus, somatic mutations in PTEN result in lack of sensitivity to androgen deprivation therapy. Abbreviations: AR - Androgen receptor; Akt - V-Akt Murine Thymoma Viral Oncogene Homolog; Chr - Chromosome; DHT - Dihydrotestosterone; K (red circle) - Kinase; PIP3 - Phosphatidylinositol (3,4,5)-Triphosphate; PTEN - Phosphatase and Tensin Homolog; T – Testosterone.

The four signaling pathways of carcinogenesis and progression affected by the somatic mutations identified in > 60% patients using this targeted panel were the phosphatidylinositol 3′-kinase (*PI3K*)-*Akt* signaling pathway (*N* = 11), mitogen-activated protein kinase signaling pathway (*MAPK*) (*N* = 11), p53 signaling pathway (*N* = 10) and Janus kinase/signal transducers and activators of transcription pathway (*JAK/STAT*) (*N* = 9) (Figure 2).

### Potential therapeutic targets

Changes potentially amenable to targeted therapy were identified in 12 (80%) cases. Of these, 11 patients expressed androgen receptor by immunohistochemistry and 6 patients demonstrated HER2 amplification by FISH. Specific mutations such as *PDGFRA* H845Y, *HRAS* Q61R, *PIK3CA* H1047R, *KIT* T670I, *EFGR* G721A and *ERBB2* V842I with potential targeted therapies were also observed in patients who had AR expression or HER2 amplification. Table 3 summarizes the genetic alterations with the potential targeted therapies. Two patients had only a *TP53* mutation, currently not amenable to targeted therapy.

**Table 3:** Analysis of SDCa for somatic mutations with potential therapeutic targets and clinical trials

Case #	Gene Symbol	Gene Name	Mutation	COSMIC ID	Functional effect	Drug
4	*PIK3CA*	Phosphatidylinositol-4,5-Bisphosphate 3-Kinase, Catalytic Subunit Alpha	c.3140A>G, p.H1047R	COSM775	Poor prognosis in breast cancer [15] Increased sensitivity to PI3K pathway inhibitors [16]	Everolimus [16], BEZ-235 (Dactolisib) [17]
11	*PIK3CA*	Phosphatidylinositol-4,5-Bisphosphate 3-Kinase, Catalytic Subunit Alpha	c.1633G>A, p.E545K	COSM763	Increased catalytic activity resulting in enhanced downstream signaling and oncogenic transformation *in vitro*[18] Associated with AKT1 activation in breast cancer [15]	NVP-BYL719 (Alpelisib) [19]
2	*PDGFRA*	Platelet Derived Growth Factor Receptor Alpha Polypeptide	c.2533C>T, H845Y	COSM96893	Increased autophosphorylation of PDGFRA [7] Responsiveness to Imatinib and Crenolanib in melanoma [7]	Imatinib, Crenolanib [7]
10	*ERBB2*	Erb-B2 Receptor Tyrosine Kinase	c.2524G>A, p.V842I	COSM14065	Increases phosphorylation of signaling proteins [8]	Lapatinib, Neratinib, Trastuzumab[8]
2	*KIT*	KIT Proto-Oncogene Receptor Tyrosine Kinase	c.2009C>T, p.T670I	COSM12708	Confers resistance to Imatinib [20] Gain of function, leads to constitutive phosphorylation of KIT [20]	Sorafenib [20]
11	*HRAS*	Harvey Rat Sarcoma Viral Oncogene Homolog	c.182A>G, p.Q61R	COSM499	Sensitized towards MEK inhibitor treatment [21]	Selumetinib and MEK162 (Binimetinib) and Everolimus [21]
4	*HRAS*	Harvey Rat Sarcoma Viral Oncogene Homolog	c.37G>C, p.G13R	COSM486	Unknown	Current Phase 1b clinical trials of MLN2480 and MLN0128 or Alisertib or Paclitaxel
1	*EGFR*	Epidermal Growth Factor Receptor	c.2162G>C, p.G721A	COSM28510	Unknown	Gefitinib, Erlotinib [22]

Specific genetic alterations were examined to assess potential suitability for targeted therapies and recorded when the functional effect and or targeted therapy had been defined in the literature.

*ongoing trial recruitment/experimental

### Somatic mutations that may alter susceptibility to androgen deprivation or HER2 inhibition therapy

**Figure 3:**
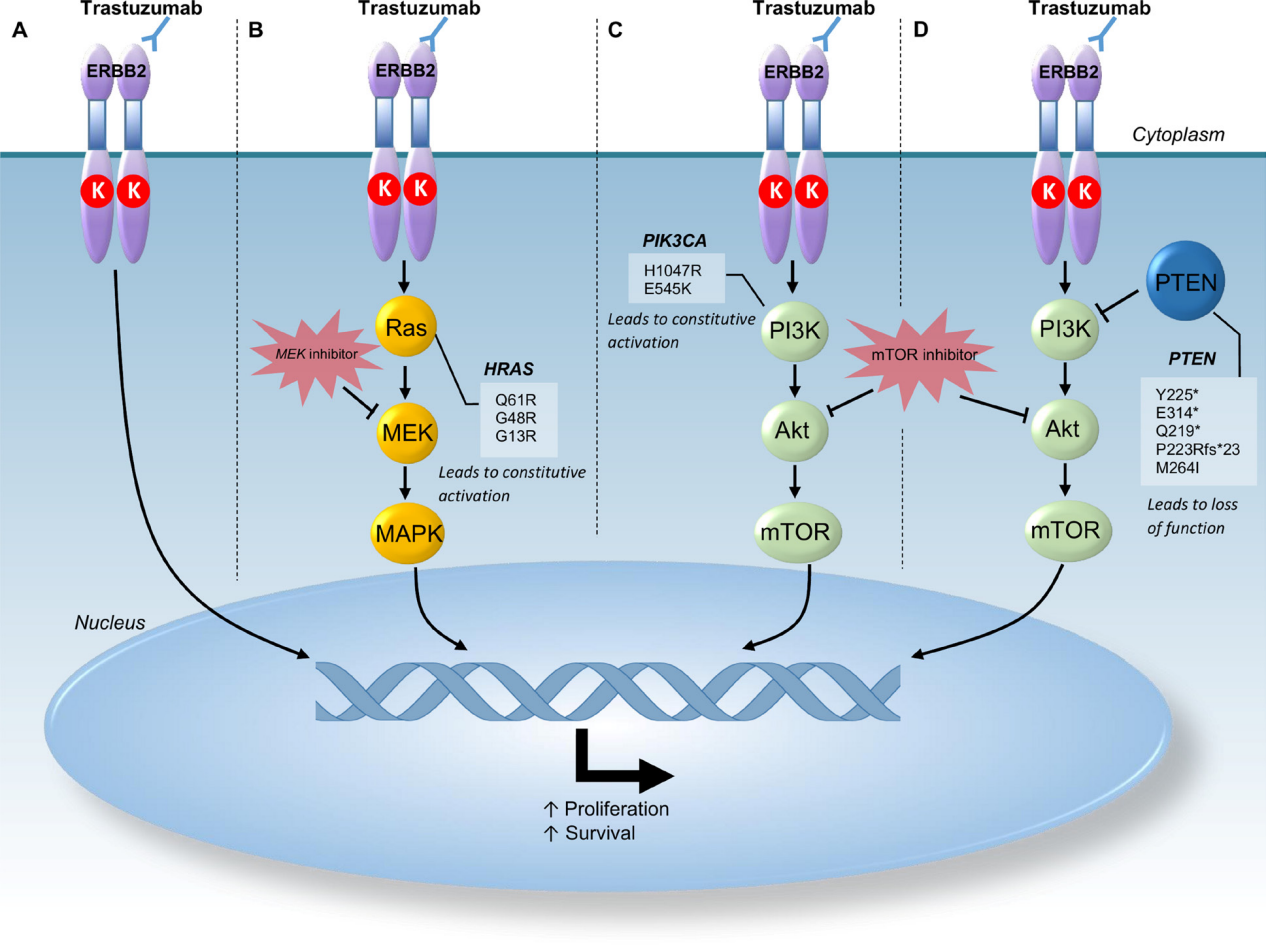
Schematic representation of HER2 signaling pathway Activation of this pathway leads to proliferation and survival of cells. Section (**A)** demonstrates normal inhibition of HER2 signaling pathway by Trastuzumab in cancers without mutations that may confer resistance. Section (**B)** demonstrates mutations in HRAS which lead to constitutive activation of the Ras gene downstream of Trastuzumab inhibition thus leading to resistance to Trastuzumab. MEK inhibitors such as Selumetinib act downstream of these activating mutations and can overcome the effects of HRAS mutations. Section (**C)** demonstrates mutations in PIK3Ca which lead to constitutive activation of this pathway downstream of Trastuzumab inhibition thus, leading to resistance of Trastuzumab therapy. Section (**D)** demonstrates mutations in PTEN that lead to truncation or a frameshift mutation resulting in a loss of function. PTEN inhibits the activation of PI3K hence, the loss of function is thought to contribute to resistance to Trastuzumab therapy. In both section C and section D a potential therapy downstream of these mutations conferring resistance to Trastuzumab include mTOR inhibitors such as Everolimus. Abbreviations: Chr - Chromosome; MAPK - Mitogen-Activated Protein Kinase; mTOR - Mechanistic Target of Rapamycin; PI3K - Phosphatidylinositol-4,5-Bisphosphonate 3-Kinase.

We also identified somatic alterations that may confer potential resistance to androgen deprivation or HER2 inhibition therapy (Table 4). *PTEN* loss as has been reported to reduce sensitivity to androgen deprivation and HER2 inhibition. This was seen in two patients with androgen receptor expression (Figures 2 and 3).

**Table 4:** Mutations conferring resistance to androgen deprivation therapy and Herceptin treatment

Case #	Gene Symbol	Gene Name	Mutation	COSMIC ID	Functional effect	Predicted Resistance
1	*PTEN*	Phosphatase and Tensin Homolog	c.675T>A, p.Y225*	COSM5291*	Truncated protein leading to predicted loss of function	Androgen Deprivation Therapy [23]; Trastuzumab [24]
2			c.940G>T, p.E314*	COSM5305		
				c.655C>T, p.Q219*			COSM5155
			c.937_940delAAGG, p.P223Rfs*23	-	Frameshift mutation resulting in a deletion/insertion	
4			c.792G>A, p.M264I	COSM5351742	Increased phosphorylation of PTEN protein, may cause configuration into inactive form [25]	
4	*PIK3CA*	Phosphatidylinositol-4,5-Bisphosphate 3-Kinase, Catalytic Subunit Alpha	c.3140A>G, p.H1047R	COSM775	Poor prognosis in breast cancer [15]Increased sensitivity to PI3K pathway inhibitors [16]	Trastuzumab [26]
11			c.1633G>A, p.E545K	COSM763	Increased catalytic activity resulting in enhanced downstream signaling and oncogenic transformation in vitro [18]Associated with AKT1 activation in breast cancer [15]	Trastuzumab [26] and Cisplatin [18]
11	*HRAS*	Harvey Rat Sarcoma Viral Oncogene Homolog	c.182A>G, p.Q61R	COSM499	May cause MAPK pathway activation [27]Interfere with GTP hydrolysis leading to hyperactive RAS kinase activity [21]	Trastuzumab and Lapatinib [4, 28]
1, 9			c.142G>A, p.G48R	COSM5555612	An activating mutation that may cause upregulation of MAPK signaling pathway [29]	
4			c.37G>C, p.G13R	COSM486	Unknown	

*COSM5291 denotes the mutation *PTEN* c.675T>G, which has the consequence of producing p.Y225* as the TAG codon is produced. The change c.675T>A produces the codon TAA which also results in a premature stop codon and truncation.

Similarly, *PIK3CA* and *HRAS* mutations that are known to reduce sensitivity to HER2 inhibition were seen in one patient that demonstrated HER2 amplification. Figure 3 illustrates the role of *PIK3CA* and *HRAS* in downstream signaling of the HER2 signaling pathway.

## DISCUSSION

Massive parallel sequencing technologies are changing the management of several common aggressive malignancies such as pulmonary adenocarcinoma and melanoma [5, 6]. Similar efforts would also benefit patients with rare malignancies such as SDCa who develop systemic metastases despite conventional therapies. The current study demonstrates that 80% of SDCa harbor alterations in pathways that may be susceptible to currently available systemic therapies, thus providing evidence towards implementation of precision medicine in these rare neoplasms. The study has also identified somatic alterations in *PIK3CA* and *HRAS* that alter sensitivity to the currently proposed therapies in SDCa such as androgen deprivation therapy and Trastuzumab. On the other hand, these alterations in *PIK3CA* and *HRAS* are susceptible to (mTOR) and mitogen extracellular signal-regulated kinases (*MEK*) inhibitors.

There are several reports of patients treated with androgen deprivation therapy, Trastuzumab and Venmurafinib for metastatic SDCa with partial to complete response [30–35]. However, these modalities have not been accepted as mainstream treatments for SDCa. Clinical trials for evaluating the effectiveness of new therapies are inherently difficult to undertake for rare malignancies [36] and the molecular pathways of carcinogenesis and progression amenable to pharmacologic modulation need to be explored. Dalin *et al.* have laid the groundwork by performing comprehensive whole exome sequencing of 16 examples of SDCa [37]. Our study demonstrates overlapping findings. Approximately 75% of patients in both studies demonstrate androgen receptor expression, 30% demonstrate HER2 amplification and nearly 70% patients harbor genetic alterations with published clinical or pre-clinical evidence supporting targeted therapies for *AR,* HER2 amplification, *PDGFRA* H845Y, *HRAS* Q61R, *PIK3CA* H1047R, *KIT* T670I and *ERBB2* V842I [7, 8, 16, 20, 21, 32, 33, 37, 38].

Clinical trials with Trastuzumab have been attempted in SDCa previously [39]. However, testing for HER2 amplification or factors that may alter response to HER2 inhibition was not undertaken prior to treatment resulting in variable response amongst the patients [39]. The findings of our study and that of Dalin *et al.* suggest that only 30–40% of patients with SDCa demonstrate HER2 amplification [37]. The experience described by Limaye *et al.* would indicate that HER2 expression is essential for response with Trastuzumab and should be undertaken prior to initiation of treatment [34]. Furthermore, the current study has also identified mutations in *PIK3CA*, *HRAS* and *PTEN* that have been shown to confer resistance to HER2 inhibition highlighting the essential role of somatic mutation testing in selecting patients for this therapy [4, 37, 40].

Our study also includes a subgroup of patients (4/15) that lack both androgen expression and HER2 amplification. Two patients also harbored both *HRAS* mutations that may be amenable to *MAPK/MEK* inhibitors such as Selumetinib [21] and *PIK3CA* H1047R mutations susceptible to mTOR inhibitors such as Everolimus [16, 41]. The coexistence of *PIK3CA* and *HRAS* mutations as observed in the current cohort is well documented in SDCa [4, 37]. These patients may benefit from therapeutic approaches using *MEK* and mTOR inhibitors synergistically [21]. Other mutations observed in isolated cases included *EGFR* G721A, which has been shown to respond to Gefinitib or Erlotinib in non-small cell lung cancer [22] and *PDGFRA* H845Y which has been shown to respond to Imatinib [7]. However, the later changes have been described in relatively small number of patients till date and warrant further investigations, particularly in SDCa. While *BRAF* V600E mutations were not identified in the current cohort, *BRAF* inhibitors such as vemurafenib may also be useful in a select group of SDCa that demonstrate these alterations as described by Nardi *et al.* [35, 42]. These findings highlight the role of somatic mutation testing in the management of SDCa.

While targeted therapies are often better tolerated than conventional chemotherapeutics, they are not without toxicities. Trastuzumab causes rare but clinically important cardiomyopathy [43]. Agents targeting the androgen pathway often cause side-effects such as sexual dysfunction, hot flushes, mood swings and fatigue [44]. Decline in bone and cardiovascular health can be seen in the long term [45]. *EGFR* inhibitors, such as Erlotinib and Gefinitib, most frequently cause skin and gastrointestinal toxicity [46]. Rational therapeutic choices based on appropriate testing, such as somatic mutation analysis is crucial to improve patient outcomes and minimize unwarranted toxicity.

It is increasingly recognized that the genotype of the tumor alone is not a reliable predictor of response to targeted therapies and transcriptomic profiling may also be useful [36]. Our approach of using formalin fixed paraffin embedded tissues from a retrospective cohort and a targeted panel is not without limitations. However, fresh tissue, ideal for genomic studies, may not always be available for rare malignancies and is less suited to clinical practice. Formalin fixed tissues generally yield short fragmented, covalently modified and cross linked DNA, but do allow analysis in the clinical setting. The findings of our study demonstrate the feasibility of using FFPE material and targeted panel sequencing. This allows for wider availability and cost effective testing providing results within a clinically relevant time frame. Analysis of FFPE tissue from a retrospective cohort with known clinical outcomes from multiple institutions can assist with development of international multi-institutional studies. Rigorous quality checks as employed in this study ensure that the results provide high quality data. Furthermore, it is encouraging that the targeted panel utilized in this study provided similar clinically relevant information as compared to the more expensive and technically challenging comprehensive methods such as whole exome sequencing. Thus, our pragmatic and cost effective approach can be widely adopted and allows for collection and development of evidence for targeted therapies in rare malignancies.

In conclusion, the majority of SDCa demonstrate molecular changes that may be amenable to pharmacologic modulations that are well established and tolerated for other cancers such as androgen deprivation therapy in prostate cancer and Trastuzumab in breast cancer [23, 26]. Testing for these molecular alterations is essential for rational therapeutic decisions. Early testing can also identify alterations that may predict resistance to these drugs. The findings of our study and that of the others provide a strong biologic argument for exploring these treatment options with pragmatically designed clinical trials [4, 37].

## MATERIALS AND METHODS

### Patients

A total of 195 cases of primary salivary gland neoplasms identified in the database of Sydney Head and Neck Cancer Institute (2000–2015) and held in the archives of the department of Tissue Pathology and Diagnostic Oncology at Royal Prince Alfred Hospital were reviewed to identify 25 cases of SDCa. Of these, suitable quality DNA extracted from FFPE tissue was available for somatic mutation testing in 15 cases after rigorous quality control. SDCa was defined as an adenocarcinoma with apocrine morphology resembling high-grade breast ductal carcinoma *in situ* (DCIS) [47]. Less common patterns include micropapillary, mucinous or sarcomatoid morphology [48]. The tumors demonstrated immunostaining for GATA3 and lacked immunoreactivity with p63 and CK 5/6. All relevant clinicopathologic information including age, gender, tumor site, tumor size, lymphovascular, perineural invasion, neck node involvement, details of local and regional failure were obtained.

All histopathologic slides were reviewed and highly cellular areas of the tumor without necrosis, stroma, inflammatory infiltrate or haemorrhage were identified. The neoplastic cell content ranged from approximately 20–80% in these areas selected for further analysis.

### Immunohistochemistry

Immunohistochemistry was performed on all cases. Androgen receptor (AR441; Dako; 1:250) staining was performed on the Leica Microsystems Bond-III automated staining platform. The Ventana BenchMark Ultra was utilized to stain HER2 (4B5; Ventana; prediluted), estrogen receptor (ER) (SP1; Ventana; prediluted), and progesterone receptor (PR) (1E2; Ventana; prediluted), as per standard protocol. The intensity of HER2 staining was graded using a widely accepted 4-point system, as defined in the literature for breast ductal carcinoma [49]. All immunohistochemistry slides were reviewed by 2 pathologists (RG and PL).

### HER2 fluorescence *in situ* hybridization

Interphase fluorescence *in-situ* hybridization (FISH) for HER2 was performed on all cases using the Vysis PathVysion HER2 DNA Probe Kit (Abbott Molecular, Des Plaines, IL) as per the manufacturer's instructions, except that Invitrogen Pretreatment Solution (Life Technologies, Carlsbad, CA) was used at 98–102°C for 20 minutes. This enumeration FISH probe quantifies HER2/neu gene copy number using a SpectrumOrange labeled 190kb DNA fragment and quantifies the chromosome 17 copy number using a SpectrumGreen labeled alpha satellite DNA fragment. Signals were counted in at least 100 tumor nuclei using an epifluorescence microscope. HER2 FISH was interpreted as per the current American Society of Clinical Oncology Guidelines for breast cancer [50].

### Somatic mutation analysis

Malignant tissue selected as described above was macrodissected from the paraffin blocks for deoxyribonucleic acid (DNA) extraction. 6–8 cores, each approximately 1mm in thickness, were taken from different areas with highest tumor cellularity. Case matched normal tissue from either the adjacent normal salivary gland parenchyma or benign neck lymph nodes was used as germline control for mutation filtering. DNA extraction was performed using truXTRAC^®^ formalin fixed paraffin embedded (FFPE) DNA microTUBE kit (Covaris, Woburn, MA, USA) as per the manufacturer**’**s instructions. Fifteen samples that had sufficient DNA that passed the quality control (QC) checks using Illumina FFPE QC Kit were used for library preparation. The TruSeq Amplification Cancer Panel^®^ (Illumina, San Diego, USA) was utilized to assess 48 clinically relevant genes: *ABL1, ALK, AKT1, APC, ATM, BRAF, CDH1, CDKN2A, CSF1R, CTNNB1, EGFR, ERBB2, ERBB4, FBXW7, FGFR1, FGFR2, FGFR3, FLT3, GNA11, GNAQ, GNAS, HNF1A, HRAS, IDH1, JAK2, JAK3, KDR, KIT, KRAS, MET, MLH1, MPL, NOTCH1, NPM1, NRAS, PDGFRA, PIK3CA, PTEN, PTPN11, RB1, RET, SMAD4, SMARCB1, SMO, SRC, STK11, TP53, VHL*. The reads were aligned to the human reference genome using Isis Smith-Waterman-Gotoh (v2.6), the variants were called using Illumina Somatic Variant Caller (v4.0) and annotated using Illumina variant studio (v2.3). Somatic variants were identified after deducting the normal/germline variants observed in the matched normal samples from those observed in the tumor samples. Variant positions with at least 500 X read depth and those variant alleles observed >5% were included in further analysis. *In-silico* analysis using PolyPhen 2, (Harvard, USA) and SIFT (J Craig Venter Institute, USA) was performed. Catalogue of Somatic Mutations in Cancer (COSMIC), Functional Analysis through Hidden Markov Models (FATHMM) for pathogenicity [51], Kyoto Encyclopedia of Genes and Genomes (KEGG) and GeneCards (Weizmann Institute of Science, Israel) databases were used for functional annotation of the identified variants and to understand their potential interactions in the relevant signal transduction pathways. The literature was extensively searched using Pubmed (English Language) for all changes identified as significant by COSMIC and FATHMM for their functional characterization in other malignancies, cell cultures or animal models. A literature search using PubMed for English language literature was performed to identify potential targetable gene variants with currently approved targeted therapies.

At an individual case level, specific genetic alterations with a described targeted therapy were sought by literature search and analyzed for potential suitability using experience from other cancers, drug availability and potential side effects.

## SUPPLEMENTARY MATERIALS TABLE




