# Supplementary material for: Somatic mutations in salivary duct carcinoma and potential therapeutic targets
Source: Oncotarget. 2017 May 25;8(44):75893–903. doi: 10.18632/oncotarget.18173 (PMC5652672; doi:10.18632/oncotarget.18173)
Supplement: Supplementary file 1 [file oncotarget-08-75893-s001.pdf]

Somatic mutations in salivary duct carcinoma and potential therapeutic targets

SUPPLEMENTARY MATERIALS

Supplementary Table 1: List of mutations occurring in cohort. See Supplementary_Table_1
