# Supplementary material for: Somatic mutations in salivary duct carcinoma and potential therapeutic targets
Source: Oncotarget. 2017 May 25;8(44):75893–903. doi: 10.18632/oncotarget.18173 (PMC5652672; doi:10.18632/oncotarget.18173)
Supplement: Supplementary file 2 [file oncotarget-08-75893-s002.doc]

| **Supplementary Table 1. List of mutations occurring in cohort** | | | | | |
| --- | --- | --- | --- | --- | --- |
| **Gene Symbol** | **Mutation** | **COSMIC ID** | **Sift** | **PolyPhen** | **HGVSc** |
| ABL1 | c.952G>T |  | deleterious(0.02) | possibly_damaging(0.824) | NM_007313.2:c.952G>T |
| ABL1 | c.944C>T |  | deleterious(0) | probably_damaging(0.98) | NM_007313.2:c.944C>T |
| ABL1 | c.944C>T |  | deleterious(0) | probably_damaging(0.98) | NM_007313.2:c.944C>T |
| ABL1 | c.1274C>T |  | deleterious(0) | probably_damaging(0.994) | NM_007313.2:c.1274C>T |
| ABL1 | c.1103C>T |  | deleterious(0.01) | possibly_damaging(0.903) | NM_007313.2:c.1103C>T |
| ABL1 | c.956A>G |  | tolerated(0.05) | probably_damaging(0.983) | NM_007313.2:c.956A>G |
| ABL1 | c.1054C>T |  |  |  | NM_007313.2:c.1054C>T |
| AKT1 | c.143G>A | COSM1368558 | tolerated(0.59) | possibly_damaging(0.715) | NM_001014431.1:c.143G>A |
| ALK | c.3776G>A |  | tolerated(0.05) | possibly_damaging(0.889) | NM_004304.4:c.3776G>A |
| APC | c.4240G>A | COSM27577 | tolerated(0.45) | benign(0.002) | NM_000038.5:c.4240G>A |
| APC | c.4016G>A | COSM143914 | tolerated(0.62) | benign(0) | NM_000038.5:c.4016G>A |
| APC | c.4414G>A | COSM18928 | tolerated(0.99) | benign(0.004) | NM_000038.5:c.4414G>A |
| APC | c.3862G>A | COSM19577 | deleterious(0.01) | possibly_damaging(0.719) | NM_000038.5:c.3862G>A |
| APC | c.4660_4661insA | COSM19695 |  |  | NM_000038.5:c.4660_4661insA |
| APC | c.4316C>T | COSM27379 | deleterious(0) | possibly_damaging(0.897) | NM_000038.5:c.4316C>T |
| APC | c.4453G>A | COSM328661 | tolerated(0.26) | benign(0.01) | NM_000038.5:c.4453G>A |
| APC | c.4630G>A |  | tolerated(0.64) | benign(0.002) | NM_000038.5:c.4630G>A |
| APC | c.4654G>A | COSM41616 | tolerated(0.87) | benign(0.001) | NM_000038.5:c.4654G>A |
| APC | c.4238T>A |  | deleterious(0) | benign(0.191) | NM_000038.5:c.4238T>A |
| APC | c.3821G>A |  | deleterious(0) | possibly_damaging(0.813) | NM_000038.5:c.3821G>A |
| APC | c.4172G>A |  | deleterious(0) | probably_damaging(0.998) | NM_000038.5:c.4172G>A |
| APC | c.4160G>A |  | deleterious(0) | probably_damaging(0.998) | NM_000038.5:c.4160G>A |
| APC | c.4208G>A |  | deleterious(0.01) | probably_damaging(0.99) | NM_000038.5:c.4208G>A |
| APC | c.4708G>A |  | deleterious(0.01) | probably_damaging(0.991) | NM_000038.5:c.4708G>A |
| APC | c.3863G>A |  | deleterious(0.02) | benign(0.329) | NM_000038.5:c.3863G>A |
| APC | c.4579C>T |  | tolerated(0.24) | possibly_damaging(0.711) | NM_000038.5:c.4579C>T |
| APC | c.4679delA |  |  |  | NM_000038.5:c.4679delA |
| ATM | c.1834C>T |  | deleterious(0.01) | probably_damaging(0.924) | NM_000051.3:c.1834C>T |
| ATM | c.2543A>G |  | deleterious(0.01) | benign(0.005) | NM_000051.3:c.2543A>G |
| ATM | c.5858C>T | COSM21625 | deleterious(0) | probably_damaging(0.997) | NM_000051.3:c.5858C>T |
| ATM (2) | c.2558C>T | COSM48858 | tolerated(0.27) | benign(0.06) | NM_000051.3:c.2558C>T |
| ATM | c.5404C>T |  | deleterious(0) | possibly_damaging(0.67) | NM_000051.3:c.5404C>T |
| ATM | c.8120C>T |  | deleterious(0) | probably_damaging(0.988) | NM_000051.3:c.8120C>T |
| ATM | c.9114G>C |  | deleterious(0) | probably_damaging(0.993) | NM_000051.3:c.9114G>C |
| ATM | c.5825C>T |  | deleterious(0) | probably_damaging(0.994) | NM_000051.3:c.5825C>T |
| ATM | c.5486C>T |  | deleterious(0) | probably_damaging(0.996) | NM_000051.3:c.5486C>T |
| ATM | c.5849C>T |  | deleterious(0) | probably_damaging(0.998) | NM_000051.3:c.5849C>T |
| ATM | c.5276C>T |  | deleterious(0.02) | benign(0.156) | NM_000051.3:c.5276C>T |
| ATM | c.5395A>T |  | tolerated(0.05) | possibly_damaging(0.592) | NM_000051.3:c.5395A>T |
| ATM | c.5840C>T |  | tolerated(0.1) | possibly_damaging(0.497) | NM_000051.3:c.5840C>T |
| ATM | c.2554C>T |  |  |  | NM_000051.3:c.2554C>T |
| BRAF | c.1378G>A | COSM1448620 | tolerated(0.06) | probably_damaging(0.999) | NM_004333.4:c.1378G>A |
| BRAF | c.1351G>A |  | deleterious(0.03) | probably_damaging(0.961) | NM_004333.4:c.1351G>A |
| BRAF | c.1379G>A |  | tolerated(0.06) | probably_damaging(0.999) | NM_004333.4:c.1379G>A |
| CDH1 | c.1223C>T | COSM143802 | deleterious(0.03) | possibly_damaging(0.8) | NM_004360.3:c.1223C>T |
| CDH1 | c.1286C>T |  | deleterious(0) | possibly_damaging(0.876) | NM_004360.3:c.1286C>T |
| CDH1 | c.1219C>T |  | tolerated(0.07) | possibly_damaging(0.789) | NM_004360.3:c.1219C>T |
| CTNNB1 | c.62C>T | COSM1422998 | deleterious(0.05) | benign(0.001) | NM_001098210.1:c.62C>T |
| EGFR | c.2162G>C | COSM28510 | deleterious(0.02) | possibly_damaging(0.848) | NM_005228.3:c.2162G>C |
| EGFR | c.2576C>T | COSM85994 | deleterious(0) | possibly_damaging(0.886) | NM_005228.3:c.2576C>T |
| EGFR | c.1874C>T |  | deleterious(0.01) | benign(0.13) | NM_005228.3:c.1874C>T |
| EGFR | c.343A>T |  | deleterious(0.02) | possibly_damaging(0.452) | NM_005228.3:c.343A>T |
| ERBB2 | c.2524G>A | COSM14065 | deleterious(0) | possibly_damaging(0.685) | NM_004448.2:c.2524G>A |
| ERBB2 | c.2600C>T |  | deleterious(0) | probably_damaging(0.99) | NM_004448.2:c.2600C>T |
| ERBB2 | c.2566C>T |  | deleterious(0) | probably_damaging(0.992) | NM_004448.2:c.2566C>T |
| ERBB2 | c.2372C>T |  | deleterious(0) | probably_damaging(0.994) | NM_004448.2:c.2372C>T |
| ERBB2 | c.2398C>T |  | deleterious(0.01) | probably_damaging(1) | NM_004448.2:c.2398C>T |
| ERBB2 | c.2578A>T |  |  |  | NM_004448.2:c.2578A>T |
| ERBB4 | c.2791G>A | COSM1405166 | tolerated(0.15) | benign(0.33) | NM_005235.2:c.2791G>A |
| ERBB4 | c.556T>C |  | deleterious(0) | probably_damaging(0.995) | NM_005235.2:c.556T>C |
| ERBB4 | c.893T>C |  | deleterious(0) | probably_damaging(0.998) | NM_005235.2:c.893T>C |
| ERBB4 | c.911G>A |  | deleterious(0) | probably_damaging(1) | NM_005235.2:c.911G>A |
| ERBB4 | c.713G>A |  | deleterious(0) | probably_damaging(1) | NM_005235.2:c.713G>A |
| ERBB4 | c.686G>A |  | deleterious(0) | probably_damaging(1) | NM_005235.2:c.686G>A |
| ERBB4 | c.397G>A |  | deleterious(0.02) | benign(0.371) | NM_005235.2:c.397G>A |
| FBXW7 | c.1322G>A | COSM1052091 | deleterious(0.03) | probably_damaging(0.978) | NM_033632.3:c.1322G>A |
| FBXW7 | c.1338G>A | COSM4167997 |  |  | NM_033632.3:c.1338G>A |
| FBXW7 | c.1172G>A | COSM1485737 | tolerated(0.61) | possibly_damaging(0.476) | NM_033632.3:c.1172G>A |
| FBXW7 | c.1318G>A | COSM22931 | deleterious(0) | probably_damaging(0.997) | NM_033632.3:c.1318G>A |
| FBXW7 | c.1429G>A | COSM327079 | deleterious(0) | probably_damaging(1) | NM_033632.3:c.1429G>A |
| FBXW7 | c.1691G>A |  | deleterious(0) | probably_damaging(0.993) | NM_033632.3:c.1691G>A |
| FBXW7 | c.1334T>G |  | deleterious(0) | probably_damaging(0.994) | NM_033632.3:c.1334T>G |
| FBXW7 | c.1333G>A |  | deleterious(0) | probably_damaging(0.994) | NM_033632.3:c.1333G>A |
| FBXW7 | c.1496G>A |  | deleterious(0) | probably_damaging(0.997) | NM_033632.3:c.1496G>A |
| FBXW7 | c.1352G>A |  | deleterious(0) | probably_damaging(0.999) | NM_033632.3:c.1352G>A |
| FBXW7 | c.1147G>A |  | deleterious(0) | probably_damaging(0.999) | NM_033632.3:c.1147G>A |
| FBXW7 | c.1670G>A |  | deleterious(0) | probably_damaging(1) | NM_033632.3:c.1670G>A |
| FBXW7 | c.1397G>A |  | deleterious(0) | probably_damaging(1) | NM_033632.3:c.1397G>A |
| FBXW7 | c.1351G>A |  | deleterious(0.01) | probably_damaging(0.989) | NM_033632.3:c.1351G>A |
| FBXW7 | c.833G>A |  | deleterious(0.02) | possibly_damaging(0.896) | NM_033632.3:c.833G>A |
| FBXW7 | c.1363C>T |  | deleterious(0.03) | benign(0.129) | NM_033632.3:c.1363C>T |
| FBXW7 | c.1648G>A |  | deleterious(0.04) | possibly_damaging(0.89) | NM_033632.3:c.1648G>A |
| FBXW7 (2) | c.1171G>A |  | tolerated(0.14) | possibly_damaging(0.713) | NM_033632.3:c.1171G>A |
| FGFR2 | c.1591G>A | COSM29833 | deleterious(0) | probably_damaging(0.996) | NM_022970.3:c.1591G>A |
| FGFR2 | c.811G>A |  | deleterious(0) | probably_damaging(0.987) | NM_022970.3:c.811G>A |
| FGFR2 | c.1608G>A |  | deleterious(0) | probably_damaging(0.998) | NM_022970.3:c.1608G>A |
| FGFR2 | c.1617delG |  |  |  | NM_022970.3:c.1617delG |
| FGFR2 | c.750G>A |  |  |  | NM_022970.3:c.750G>A |
| FGFR3 | c.779C>T |  | deleterious(0) | probably_damaging(0.965) | NM_001163213.1:c.779C>T |
| FGFR3 | c.1195C>T |  | deleterious(0) | probably_damaging(0.982) | NM_001163213.1:c.1195C>T |
| FGFR3 | c.2086C>A |  | deleterious(0) | probably_damaging(0.986) | NM_001163213.1:c.2086C>A |
| FGFR3 | c.2104G>T |  | deleterious(0.01) | possibly_damaging(0.571) | NM_001163213.1:c.2104G>T |
| FGFR3 | c.862C>T |  | deleterious(0.04) | possibly_damaging(0.454) | NM_001163213.1:c.862C>T |
| FGFR3 | c.2167C>T |  | tolerated(0.12) | possibly_damaging(0.788) | NM_001163213.1:c.2167C>T |
| FGFR3 | c.1968C>T |  |  |  | NM_001163213.1:c.1968C>T |
| FGFR3 | c.766C>T |  |  |  | NM_001163213.1:c.766C>T |
| FLT3 | c.1352C>T | COSM28042 | deleterious(0) | probably_damaging(0.947) | NM_004119.2:c.1352C>T |
| FLT3 | c.1316C>T |  | deleterious(0) | probably_damaging(0.947) | NM_004119.2:c.1316C>T |
| FLT3 | c.1810G>A |  | deleterious(0) | probably_damaging(0.996) | NM_004119.2:c.1810G>A |
| FLT3 | c.2497G>A |  | deleterious(0) | probably_damaging(0.999) | NM_004119.2:c.2497G>A |
| FLT3 | c.2437G>A |  | deleterious(0) | probably_damaging(0.999) | NM_004119.2:c.2437G>A |
| FLT3 | c.2036G>A |  | deleterious(0) | probably_damaging(1) | NM_004119.2:c.2036G>A |
| FLT3 | c.2449G>A |  | deleterious(0.01) | probably_damaging(0.949) | NM_004119.2:c.2449G>A |
| FLT3 | c.1748G>A |  | deleterious(0.02) | probably_damaging(0.997) | NM_004119.2:c.1748G>A |
| FLT3 | c.1367G>C |  | deleterious(0.03) | possibly_damaging(0.708) | NM_004119.2:c.1367G>C |
| FLT3 | c.2473G>A |  | tolerated(0.12) | possibly_damaging(0.533) | NM_004119.2:c.2473G>A |
| FLT3 | c.1970delC |  |  |  | NM_004119.2:c.1970delC |
| FLT3 | c.1345C>T |  |  |  | NM_004119.2:c.1345C>T |
| GNA11 | c.618delG | COSM1392334 |  |  | NM_002067.2:c.618delG |
| GNA11 | c.1015T>C |  | deleterious(0.02) | benign(0.011) | NM_002067.2:c.1015T>C |
| GNA11 | c.583_584delGA |  |  |  | NM_002067.2:c.583_584delGA |
| GNAQ (2) | c.890G>A |  | deleterious(0) | probably_damaging(0.976) | NM_002072.3:c.890G>A |
| GNAQ | c.752G>A |  | deleterious(0) | probably_damaging(0.982) | NM_002072.3:c.752G>A |
| GNAQ | c.694C>T |  | deleterious(0) | probably_damaging(0.985) | NM_002072.3:c.694C>T |
| GNAQ | c.1012C>T |  | deleterious(0) | probably_damaging(0.987) | NM_002072.3:c.1012C>T |
| GNAQ | c.749A>T |  | deleterious(0) | probably_damaging(0.993) | NM_002072.3:c.749A>T |
| GNAQ | c.652C>T |  | deleterious(0) | probably_damaging(0.997) | NM_002072.3:c.652C>T |
| GNAQ | c.857C>T |  | deleterious(0.01) | probably_damaging(0.999) | NM_002072.3:c.857C>T |
| GNAQ | c.493G>A |  | deleterious(0.03) | benign(0.324) | NM_002072.3:c.493G>A |
| GNAQ | c.862C>A |  | deleterious(0.04) | benign(0.264) | NM_002072.3:c.862C>A |
| GNAQ | c.935T>C |  | tolerated(0.23) | possibly_damaging(0.439) | NM_002072.3:c.935T>C |
| GNAQ | c.605G>A |  | tolerated(0.4) | possibly_damaging(0.856) | NM_002072.3:c.605G>A |
| GNAS | c.2527T>C |  | deleterious(0) | probably_damaging(1) | NM_080425.2:c.2527T>C |
| HNF1A | c.911C>T |  | deleterious(0.02) | probably_damaging(0.909) | NM_000545.5:c.911C>T |
| HNF1A | c.844G>A |  | deleterious(0.03) | probably_damaging(0.998) | NM_000545.5:c.844G>A |
| HNF1A | c.940C>T |  | tolerated(0.27) | possibly_damaging(0.471) | NM_000545.5:c.940C>T |
| HRAS | c.182A>G | COSM499 | deleterious(0.02) | benign(0.125) | NM_005343.2:c.182A>G |
| HRAS | c.37G>C | COSM486 | deleterious(0) | probably_damaging(0.996) | NM_005343.2:c.37G>C |
| HRAS (2) | c.142G>A |  | deleterious(0) | probably_damaging(0.989) | NM_005343.2:c.142G>A |
| HRAS | c.40G>A |  | deleterious(0) | probably_damaging(0.997) | NM_005343.2:c.40G>A |
| HRAS | c.152G>A |  | tolerated(0.08) | possibly_damaging(0.685) | NM_005343.2:c.152G>A |
| HRAS | c.154C>A |  | tolerated(0.45) | possibly_damaging(0.754) | NM_005343.2:c.154C>A |
| IDH1 | c.341G>A |  | deleterious(0) | probably_damaging(0.973) | NM_005896.2:c.341G>A |
| IDH1 | c.326G>A |  | deleterious(0) | probably_damaging(0.999) | NM_005896.2:c.326G>A |
| IDH1 | c.342delC |  |  |  | NM_005896.2:c.342delC |
| JAK3 | c.2148G>A |  |  |  | NM_000215.3:c.2148G>A |
| KDR (2) | c.1416A>T | COSM149673 | tolerated(0.1) | benign(0.012) | NM_002253.2:c.1416A>T |
| KDR | c.2617G>A | COSM32294 | deleterious(0) | probably_damaging(1) | NM_002253.2:c.2617G>A |
| KDR | c.716G>A |  | deleterious(0) | probably_damaging(1) | NM_002253.2:c.716G>A |
| KDR | c.673G>A |  | tolerated(0.06) | possibly_damaging(0.837) | NM_002253.2:c.673G>A |
| KDR | c.712G>A |  | tolerated(0.07) | possibly_damaging(0.645) | NM_002253.2:c.712G>A |
| KDR | c.2932G>A |  | tolerated(0.12) | probably_damaging(0.906) | NM_002253.2:c.2932G>A |
| KDR | c.2948T>C |  | tolerated(0.15) | probably_damaging(0.973) | NM_002253.2:c.2948T>C |
| KDR | c.2857G>A |  | tolerated(0.4) | probably_damaging(0.996) | NM_002253.2:c.2857G>A |
| KDR | c.3623G>A |  | tolerated(0.52) | possibly_damaging(0.708) | NM_002253.2:c.3623G>A |
| KDR | c.4021C>T |  |  |  | NM_002253.2:c.4021C>T |
| KIT | c.2009C>T | COSM12708 | deleterious(0) | probably_damaging(1) | NM_000222.2:c.2009C>T |
| KIT | c.1621A>C | COSM28026 | tolerated(0.34) | benign(0.008) | NM_000222.2:c.1621A>C |
| KIT | c.2084A>G | COSM1430165 | tolerated(0.18) | benign(0.339) | NM_000222.2:c.2084A>G |
| KIT | c.2512C>T |  | deleterious(0) | probably_damaging(1) | NM_000222.2:c.2512C>T |
| KIT | c.1717C>T |  | deleterious(0.01) | possibly_damaging(0.767) | NM_000222.2:c.1717C>T |
| KIT | c.2201C>T |  | deleterious(0.04) | possibly_damaging(0.846) | NM_000222.2:c.2201C>T |
| KIT | c.1678G>C |  | deleterious(0.05) | benign(0.307) | NM_000222.2:c.1678G>C |
| KRAS | c.19G>A | COSM30620 | deleterious(0) | probably_damaging(1) | NM_033360.2:c.19G>A |
| KRAS | c.413G>A |  | deleterious(0.03) | probably_damaging(0.911) | NM_033360.2:c.413G>A |
| KRAS | c.122G>A |  | tolerated(0.06) | possibly_damaging(0.441) | NM_033360.2:c.122G>A |
| KRAS | c.384delA |  |  |  | NM_033360.2:c.384delA |
| MET | c.3082+2T>C | COSM35468 |  |  | NM_001127500.1:c.3082+2T>C |
| MET | c.3821A>G |  | deleterious(0) | probably_damaging(0.967) | NM_001127500.1:c.3821A>G |
| MET (2) | c.3845C>T |  | deleterious(0) | probably_damaging(1) | NM_001127500.1:c.3845C>T |
| MET | c.540_541delGG |  |  |  | NM_001127500.1:c.540_541delGG |
| MLH1 | c.1153C>T | COSM1422593 | deleterious(0) | probably_damaging(1) | NM_000249.3:c.1153C>T |
| MPL | c.1565+5_1565+6delCG |  |  |  | NM_005373.2:c.1565+5_1565+6delCG |
| NOTCH1 | c.4793G>A | COSM5991555 | tolerated(0.76) | benign(0.031) | NM_017617.3:c.4793G>A |
| NOTCH1 | c.5026G>A | COSM327182 | tolerated(0.09) | probably_damaging(0.997) | NM_017617.3:c.5026G>A |
| NOTCH1 | c.4723G>A |  | deleterious(0.01) | probably_damaging(0.972) | NM_017617.3:c.4723G>A |
| NOTCH1 | c.4795G>A |  | tolerated(0.1) | probably_damaging(0.914) | NM_017617.3:c.4795G>A |
| NPM1 | c.855A>T |  | deleterious(0.04) | benign(0.044) | NM_002520.6:c.855A>T |
| NRAS | c.145G>A | COSM14199 | tolerated(0.14) | benign(0.111) | NM_002524.4:c.145G>A |
| NRAS | c.43G>A | COSM1332937 | deleterious(0) | probably_damaging(0.991) | NM_002524.4:c.43G>A |
| NRAS | c.50G>A | COSM253332 | deleterious(0) | probably_damaging(0.996) | NM_002524.4:c.50G>A |
| NRAS | c.179G>A | COSM28673 | deleterious(0) | probably_damaging(1) | NM_002524.4:c.179G>A |
| NRAS | c.169G>A |  | deleterious(0) | probably_damaging(0.979) | NM_002524.4:c.169G>A |
| NRAS | c.143G>A |  | tolerated(0.32) | possibly_damaging(0.613) | NM_002524.4:c.143G>A |
| NRAS | c.22delG |  |  |  | NM_002524.4:c.22delG |
| PDGFRA | c.2533C>T | COSM96893 | deleterious(0.02) | probably_damaging(0.968) | NM_006206.4:c.2533C>T |
| PDGFRA | c.1755A>T | COSM96949 | tolerated(0.8) | probably_damaging(0.946) | NM_006206.4:c.1755A>T |
| PDGFRA | c.1994C>T |  | deleterious(0) | probably_damaging(1) | NM_006206.4:c.1994C>T |
| PIK3CA | c.277C>T | COSM27493 | deleterious(0) | probably_damaging(0.995) | NM_006218.2:c.277C>T |
| PIK3CA | c.333G>C | COSM12580 | deleterious(0) | probably_damaging(0.947) | NM_006218.2:c.333G>C |
| PIK3CA | c.1633G>A | COSM763 | deleterious(0.01) | possibly_damaging(0.868) | NM_006218.2:c.1633G>A |
| PIK3CA | c.3152G>A | COSM308549 |  |  | NM_006218.2:c.3152G>A |
| PIK3CA | c.3149G>A | COSM17446 | tolerated(0.1) | benign(0.352) | NM_006218.2:c.3149G>A |
| PIK3CA | c.3140A>G | COSM775 | tolerated(0.36) | benign(0.205) | NM_006218.2:c.3140A>G |
| PIK3CA | c.3059C>T | COSM28938 | tolerated(0.25) | possibly_damaging(0.6) | NM_006218.2:c.3059C>T |
| PIK3CA | c.3016C>T | COSM328022 | deleterious(0) | probably_damaging(0.995) | NM_006218.2:c.3016C>T |
| PIK3CA | c.290C>T |  | deleterious(0) | possibly_damaging(0.743) | NM_006218.2:c.290C>T |
| PIK3CA | c.2132A>G |  | deleterious(0) | possibly_damaging(0.864) | NM_006218.2:c.2132A>G |
| PIK3CA | c.340A>T |  | deleterious(0) | probably_damaging(0.963) | NM_006218.2:c.340A>T |
| PIK3CA | c.271G>T |  | deleterious(0) | probably_damaging(0.997) | NM_006218.2:c.271G>T |
| PIK3CA | c.3178C>G |  | deleterious(0) | probably_damaging(0.998) | NM_006218.2:c.3178C>G |
| PIK3CA (2) | c.257C>T |  | deleterious(0.02) | possibly_damaging(0.667) | NM_006218.2:c.257C>T |
| PIK3CA | c.310C>T |  | tolerated(0.11) | probably_damaging(0.92) | NM_006218.2:c.310C>T |
| PIK3CA | c.2101C>T |  | tolerated(0.59) | possibly_damaging(0.624) | NM_006218.2:c.2101C>T |
| PTEN | c.1004G>A | COSM1349620 | deleterious(0.02) | benign(0.291) | NM_000314.4:c.1004G>A |
| PTEN (2) | c.638C>T |  | tolerated(0.86) | benign(0.158) | NM_000314.4:c.638C>T |
| PTEN | c.940G>T | COSM5305 |  |  | NM_000314.4:c.940G>T |
| PTEN | c.724G>A | COSM5087 | tolerated(0.49) | benign(0.039) | NM_000314.4:c.724G>A |
| PTEN | c.655C>T | COSM5155 |  |  | NM_000314.4:c.655C>T |
| PTEN | c.675T>A | COSM5291 |  |  | NM_000314.4:c.675T>A |
| PTEN | c.39A>T |  | deleterious(0.01) | possibly_damaging(0.768) | NM_000314.4:c.39A>T |
| PTEN | c.629C>T |  | tolerated(0.08) | possibly_damaging(0.885) | NM_000314.4:c.629C>T |
| PTEN | c.557T>A |  | tolerated(0.13) | possibly_damaging(0.828) | NM_000314.4:c.557T>A |
| PTEN (2) | c.605C>T |  | tolerated(0.14) | probably_damaging(0.994) | NM_000314.4:c.605C>T |
| PTEN | c.586C>T |  | tolerated(0.65) | possibly_damaging(0.891) | NM_000314.4:c.586C>T |
| PTEN | c.937_940delAAGG |  |  |  | NM_000314.4:c.937_940delAAGG |
| PTPN11 | c.302C>T |  | deleterious(0) | possibly_damaging(0.892) | NM_002834.3:c.302C>T |
| PTPN11 | c.292C>T |  | deleterious(0.01) | probably_damaging(0.997) | NM_002834.3:c.292C>T |
| PTPN11 | c.235C>T |  |  |  | NM_002834.3:c.235C>T |
| RB1 | c.1786C>T |  | tolerated(0.12) | benign(0.06) | NM_000321.2:c.1786C>T |
| RB1 | c.1727C>T | COSM13140 | tolerated(0.13) | benign(0.035) | NM_000321.2:c.1727C>T |
| RB1 | c.1072C>T | COSM879 |  |  | NM_000321.2:c.1072C>T |
| RB1 | c.2315C>T |  | deleterious(0.01) | probably_damaging(0.953) | NM_000321.2:c.2315C>T |
| RB1 (2) | c.1789C>T |  |  |  | NM_000321.2:c.1789C>T |
| RB1 | c.397delA |  |  |  | NM_000321.2:c.397delA |
| RET | c.1891G>A | COSM1237916 | tolerated(0.2) | benign(0.372) | NM_020975.4:c.1891G>A |
| RET (2) | c.2354C>T |  | deleterious(0) | probably_damaging(1) | NM_020975.4:c.2354C>T |
| RET | c.2321C>T |  | deleterious(0) | probably_damaging(1) | NM_020975.4:c.2321C>T |
| RET (2) | c.2353C>T |  | deleterious(0.01) | probably_damaging(1) | NM_020975.4:c.2353C>T |
| RET | c.2772T>G |  | tolerated(0.08) | possibly_damaging(0.875) | NM_020975.4:c.2772T>G |
| RET | c.2309G>T |  | tolerated(0.13) | probably_damaging(0.946) | NM_020975.4:c.2309G>T |
| RET | c.2667_2673delGATTTCG |  |  |  | NM_020975.4:c.2667_2673delGATTTCG |
| SMAD4 | c.737C>T | COSM1226728 | tolerated(0.64) | benign(0.002) | NM_005359.5:c.737C>T |
| SMAD4 (3) | c.1028C>T |  | deleterious(0) | probably_damaging(0.938) | NM_005359.5:c.1028C>T |
| SMAD4 | c.733C>T | COSM14057 |  |  | NM_005359.5:c.733C>T |
| SMAD4 | c.608C>T | COSM14180 | tolerated(0.06) | benign(0.008) | NM_005359.5:c.608C>T |
| SMAD4 | c.1096C>T | COSM30781 |  |  | NM_005359.5:c.1096C>T |
| SMAD4 | c.1037C>T |  | deleterious(0) | probably_damaging(0.905) | NM_005359.5:c.1037C>T |
| SMAD4 | c.1361C>T |  | deleterious(0.01) | benign(0.433) | NM_005359.5:c.1361C>T |
| SMAD4 | c.1036C>T |  | tolerated(0.13) | possibly_damaging(0.702) | NM_005359.5:c.1036C>T |
| SMAD4 | c.1378delG |  |  |  | NM_005359.5:c.1378delG |
| SMO | c.1025C>T |  | deleterious(0) | probably_damaging(0.946) | NM_005631.4:c.1025C>T |
| SMO | c.1007C>T |  | deleterious(0) | probably_damaging(0.962) | NM_005631.4:c.1007C>T |
| SMO | c.1934C>T |  | deleterious(0.01) | probably_damaging(0.995) | NM_005631.4:c.1934C>T |
| SMO | c.1053G>T |  | deleterious(0.03) | probably_damaging(0.907) | NM_005631.4:c.1053G>T |
| SMO | c.1043C>T |  | deleterious(0.03) | probably_damaging(0.924) | NM_005631.4:c.1043C>T |
| SMO | c.1933C>T |  | deleterious(0.03) | probably_damaging(0.994) | NM_005631.4:c.1933C>T |
| SRC | c.1594C>A |  | deleterious(0.01) | possibly_damaging(0.776) | NM_005417.3:c.1594C>A |
| STK11 | c.166G>C | COSM48784 | deleterious(0) | probably_damaging(1) | NM_000455.4:c.166G>C |
| STK11 | c.186G>T |  | deleterious(0) | probably_damaging(0.964) | NM_000455.4:c.186G>T |
| STK11 | c.140G>C |  | deleterious(0.02) | possibly_damaging(0.528) | NM_000455.4:c.140G>C |
| STK11 | c.862G>A |  | tolerated(0.19) | probably_damaging(0.929) | NM_000455.4:c.862G>A |
| TP53 | c.524G>A | COSM10648 | tolerated(0.13) | benign(0.209) | NM_000546.5:c.524G>A |
| TP53 | c.742C>T | COSM10656 | deleterious(0) | probably_damaging(1) | NM_000546.5:c.742C>T |
| TP53 | c.916C>T | COSM10663 |  |  | NM_000546.5:c.916C>T |
| TP53 | c.577C>T | COSM10672 | deleterious(0) | probably_damaging(1) | NM_000546.5:c.577C>T |
| TP53 | c.548C>G | COSM10706 |  |  | NM_000546.5:c.548C>G |
| TP53 | c.856G>A | COSM10726 | deleterious(0) | probably_damaging(0.987) | NM_000546.5:c.856G>A |
| TP53 | c.438G>A | COSM10727 |  |  | NM_000546.5:c.438G>A |
| TP53 | c.310C>A |  | deleterious(0.03) | benign(0.028) | NM_000546.5:c.310C>A |
| TP53 | c.536A>C | COSM44218 | deleterious(0.01) | possibly_damaging(0.452) | NM_000546.5:c.536A>C |
| TP53 | c.580C>T | COSM10995 | deleterious(0) | probably_damaging(1) | NM_000546.5:c.580C>T |
| TP53 | c.799C>T | COSM11183 | deleterious(0.03) | possibly_damaging(0.72) | NM_000546.5:c.799C>T |
| TP53 | c.497C>T | COSM44289 | tolerated(0.08) | benign(0.151) | NM_000546.5:c.497C>T |
| TP53 | c.376-1G>A | COSM18655 |  |  | NM_000546.5:c.376-1G>A |
| TP53 | c.376T>C | COSM4435840 | deleterious(0) | probably_damaging(1) | NM_000546.5:c.376T>C |
| TP53 | c.431A>C | COSM44205 | deleterious(0.01) | probably_damaging(0.998) | NM_000546.5:c.431A>C |
| TP53 | c.232G>A | COSM219129 | tolerated(0.56) | benign(0.005) | NM_000546.5:c.232G>A |
| TP53 | c.427G>A | COSM43878 | deleterious(0) | probably_damaging(0.998) | NM_000546.5:c.427G>A |
| TP53 | c.1025G>A | COSM45278 | tolerated(0.23) | benign(0.022) | NM_000546.5:c.1025G>A |
| TP53 | c.908G>A | COSM43986 | tolerated(0.2) | benign(0.022) | NM_000546.5:c.908G>A |
| TP53 | c.389T>A | COSM46114 | deleterious(0) | probably_damaging(1) | NM_000546.5:c.389T>A |
| TP53 | c.399G>A | COSM44624 | deleterious(0.01) | benign(0.012) | NM_000546.5:c.399G>A |
| TP53 | c.385G>A | COSM44966 | tolerated(0.57) | benign(0.001) | NM_000546.5:c.385G>A |
| TP53 (2) | c.215C>G | COSM45985 | tolerated(0.56) | benign(0.033) | NM_000546.5:c.215C>G |
| TP53 | c.668_671delCTGA |  |  |  | NM_000546.5:c.668_671delCTGA |
| VHL | c.575C>T | COSM14353 | deleterious(0.01) | probably_damaging(1) | NM_000551.3:c.575C>T |
| VHL | c.539T>A | COSM18000 | deleterious(0) | probably_damaging(1) | NM_000551.3:c.539T>A |
| VHL | c.340+8C>T | COSM18055 |  |  | NM_000551.3:c.340+8C>T |
| VHL | c.332G>C |  | tolerated(1) | benign(0.003) | NM_000551.3:c.332G>C |
| VHL | c.328C>T |  | deleterious(0.04) | possibly_damaging(0.74) | NM_000551.3:c.328C>T |
| VHL | c.487C>T |  | deleterious(0.05) | probably_damaging(0.998) | NM_000551.3:c.487C>T |
| VHL | c.319C>T |  | tolerated(0.19) | possibly_damaging(0.875) | NM_000551.3:c.319C>T |
| VHL | c.573delC |  |  |  | NM_000551.3:c.573delC |
| Mutations occurring multiple times are denoted with (x), where x = frequency of mutation | | | | | |
